# A rare presentation of ectopic ovary in a female adolescent and the impact of obesity: a case report

**DOI:** 10.1186/s13256-016-1084-3

**Published:** 2016-10-19

**Authors:** Valirie Ndip Agbor, Tsi Njim, Leopold Ndemnge Aminde

**Affiliations:** 1 Hope Clinic Bamenda, Bamenda, North-West Region Cameroon; 2 Nuffield Department of Medicine, University of Oxford, Oxford, Oxfordshire UK; 3 Health and Human Development (2HD) Research Group, Douala, Cameroon; 4 Clinical Research Education, Networking and Consultancy (CRENC), Douala, Littoral Cameroon; 5 School of Public Health, Faculty of Medicine & Biomedical Sciences, University of Queensland, Brisbane, QLD 4006 Australia

**Keywords:** Ectopic ovary, Inguinal hernia, Obesity, Cameroon

## Abstract

**Background:**

Inguinal hernias in women of reproductive age containing the ovary are very rare. When they occur in this age group, they are mostly associated with malformations of the urogenital system. Prompt surgical intervention is the key to ensure survival of the ovary. Here we present a case of an ectopic ovary presenting like an acute appendicitis.

**Case presentation:**

A 16-year-old Cameroonian girl presented at our emergency service with an acute exacerbation of a mild and intermittent right iliac fossa pain of 5 days’ duration. A clinical examination revealed android obesity and signs suggestive of an acute appendicitis. An abdominopelvic ultrasound scan showed an edematous right ovary in the canal of Nuck. A prompt hernia repair was done and her postoperative period was uneventful.

**Conclusions:**

An ectopic inguinal ovary remains a rare occurrence. An urgent and careful exploration of the hernia sac is the standard of care. Careful physical examination of obese girls and women is vital particularly in emergency settings, as obesity in our patient contributed greatly to a missed diagnosis. Clinicians should potentially consider the possibility of an ectopic ovary when faced with girls and women presenting with right iliac fossa pain.

## Background

Inguinal hernias occur in less than 5 % of women. These hernias usually contain a variety of viscera, commonly the intestines and omentum. An inguinal hernia containing the ovary is very rare in women of reproductive age. Only 3 % of cases of inguinal hernias contain viscera such as female adnexa [1], 30 % of which occur in adolescents or women of reproductive age [2]. In most cases, the diagnosis of a hernia is clinical; however, an ultrasound scan is helpful in uncertain circumstances. Inguinal hernias can present either as asymptomatic progressive groin swellings or acutely, with associated mild to severe abdominopelvic pain [3]. We report the case of a 16-year-old girl with an incarcerated right ovarian inguinal hernia presenting like acute appendicitis.

## Case presentation

A 16-year-old Cameroonian girl presented to our emergency service with an acute exacerbation of a mild and intermittent right iliac fossa (RIF) pain of 5 days’ duration associated with anorexia and nausea. Her last menstrual period was 2 weeks prior to consultation. Her menarche was at the age of 12 years and she had a menstrual flow of 3 to 4 days with a regular cycle of 28 to 30 days. She was neither sexually active nor had a history of dysmenorrhea, and the rest of her past history was unremarkable. There was no vomiting, obstipation, fever, abnormal vaginal discharges, or urinary tract symptoms. On clinical examination, she was anxious with pain quoted 6/10 according to the visual analog pain scale, tachycardic (pulse rate of 110 beats per minute), and afebrile (maximum temperature of 36.8 °C). Her body mass index (BMI) was 34.2 kg/m^2^ and waist circumference 101 cm. She had tenderness at the McBurney’s point, and positive Bloomberg, obturator, and psoas signs. The clinical presentation suggested a presumptive diagnosis of acute appendicitis.

**Fig. 1:**
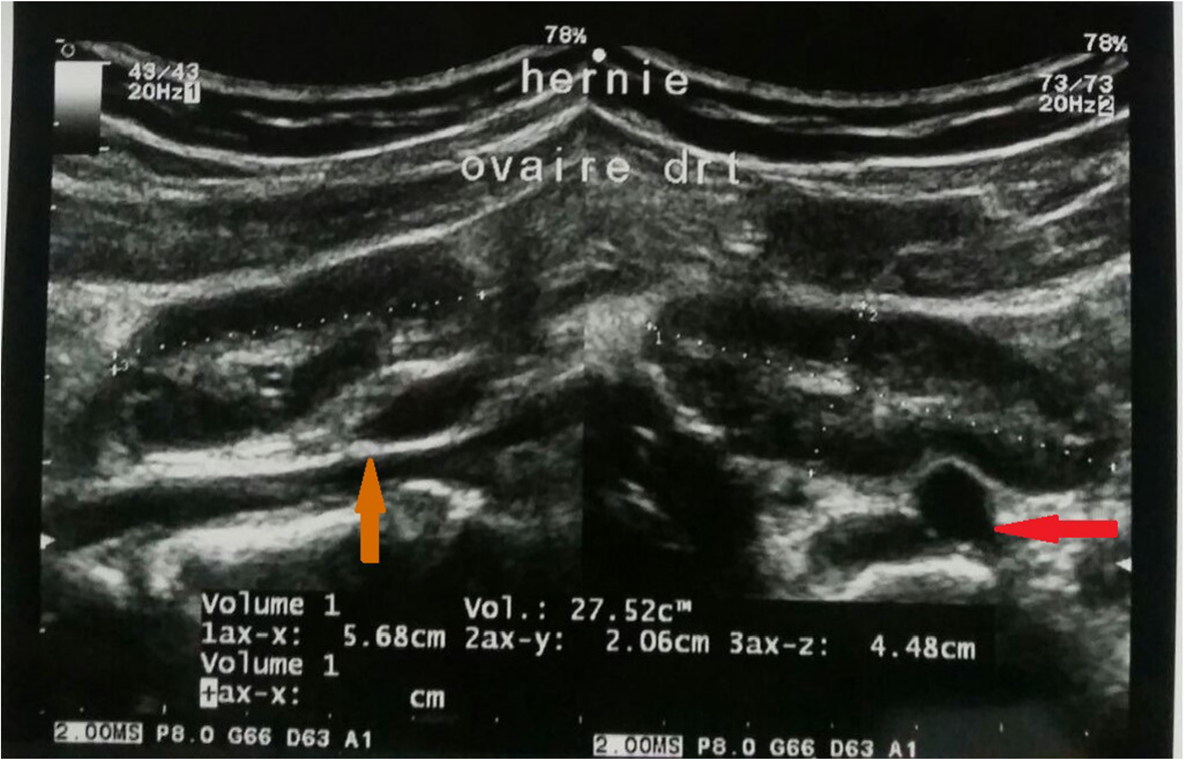
Pelvic ultrasound showing an edematous right ovary in longitudinal (left) and transverse (right) views. On the *left*, the ovary is superficial to the parietal peritoneum (*orange arrow*), and just beneath the subcutaneous tissues. On the *right*, the ovary is superficial to the external iliac artery (*red arrow*)

A complete blood count (CBC) with differentials, C-reactive protein (CRP), and a urine analysis were normal, and her pregnancy test was negative. An abdominopelvic ultrasound scan revealed an edematous right ovary measuring 5.7×2.1×4.5 cm, in the canal of Nuck (clearly visible in real-time motion; Fig. 1). We therefore concluded on the diagnosis of an incarcerated right inguinal hernia of the ovary. An urgent gynecological review was made and she was immediately prepared for surgery. The timeframe from diagnosis to surgery was approximately 2 hours and 30 minutes. An incarcerated, edematous, but viable right ovary was found intraoperatively in her canal of Nuck. Her right fallopian tube, her uterus, and left adnexa were macroscopically normal. Her left internal inguinal ring was intact and her appendix was not inflamed. A right inguinal herniorrhaphy using the Bassini technique was done and the deep inguinal ring reinforced. Her postoperative period was uneventful and there were no new complaints after 6 months of follow up.

## Discussion

In this case report, we highlight a rare presentation of an ectopic ovary in an adolescent girl and the adverse contribution of obesity. Besides the ovary located in the canal of Nuck, and features rather suggestive of acute appendicitis, the initial misdiagnosis in part was potentially due to her increased waistline and android obesity. There are two types of groin hernias: inguinal hernias that constitute approximately 96 % of these hernias, and femoral hernias which make up just 4 % of groin hernias. Inguinal hernias are nine times more frequent in boys than in girls. Worldwide, inguinal hernia repair is one of the most frequently performed surgical procedures [4]. Even though inguinal hernias are relatively common, only 3 % contain viscera such as the female adnexa, 30 % of which are reported to occur in adolescents or women of reproductive age [2].

The canal of Nuck described by the Dutch anatomist in 1691, is a virtual space formed by an abnormal patent pouch of peritoneum and is the female analogue of the processus vaginalis in the male. Normally, during embryonic life, the parietal peritoneum evaginates accompanying the round ligament of the uterus through the inguinal canal to insert at the base of the labia majora on the ipsilateral side. This peritoneal evagination normally obliterates around the eighth month of intrauterine life [5]. The internal inguinal ring normally closes following migration of the distal gubernaculum through the inguinal canal and into the labia majora. The ovary is prevented from descending to the base of the labia majora by fixation of the proximal gubernaculum of the ovary to the cornu of the uterus.

A persistence of the canal of Nuck, coupled with either a failure of closure of the internal ring or attachment of the proximal gubernaculum to the uterine cornu, and/or an abnormally short distal gubernaculum provides the defect necessary for the development of an ectopic inguinal ovary [6]. Also, the ovary may adhere to the mesentery and/or bowel, subsequently forming part of the contents of an inguinal hernia sac [7]. Factors which will cause a persistent increase in intra-abdominal pressure such as pregnancy, chronic bronchitis, whooping cough, and frequent heavy lifting do favor the occurrence of ectopic inguinal ovaries [6].

Pain and tenderness on the affected side during menstruation is usually the chief complaint of patients with an ectopic inguinal ovary. Mette and colleagues described a case of an adult woman who presented with pelvic pain on a longstanding history of left iliac fossa pain [8]. Palpation usually reveals a lump in the groin, oval in shape, firm, mobile, and which neither pulsates nor gives a positive cough impulse. Our patient presented with features suggestive of an acute appendicitis, and it was difficult to appreciate the groin mass due to her significant android obesity. The index case thus highlights the need for clinicians to be meticulous during clinical examination of individuals who are overweight and obese. The absence of pain during her previous menstruations also suggested an acute event. According to Mayer and Templeton, strangulation was the most common complication of a herniated inguinal ovary, even though cystic degeneration and malignant transformations of the ovary were also potential complications [7]. Our patient presented with incarceration, similar to reports elsewhere among young babies [9].

The diagnosis of an ectopic inguinal ovary is based on the history and clinical examination. In cases of doubt, ultrasonography with high-frequency transducers is the examination of choice to establish a definitive diagnosis [10]. A MEDLINE search of case reports revealed that a majority of cases with an ectopic inguinal ovary were diagnosed during the course of surgery indicated for an intestinal loop groin hernia [11]. A preoperative ultrasound scan played a vital role in establishing the diagnosis of an ectopic inguinal ovary in our case. Ectopic inguinal ovaries are usually associated with urinary malformations [6], or internal genital malformations, when occurring in women of reproductive age [12]. There is limited published data on patients presenting with ectopic inguinal ovaries and normally developed urinary and genital system, as was observed in our patient [9].

Acute RIF pain in women of reproductive age can be attributed to a variety of causes depending on the affected system: gynecologic (acute salpingitis, torsion or hemorrhagic ovarian cyst, tubo-ovarian abscess, and ectopic pregnancy), gastrointestinal (acute appendicitis, mesenteric adenitis, terminal ileitis, diverticulitis, Crohn’s disease, and gastroenteritis), and urologic (urinary tract infection and renal colic). RIF pain due to ectopic ovary is relatively inconsistent with existing literature.

The use of prosthetic materials for inguinal hernia repair yields the best results according to data from Europe and the USA [4]. However, these materials are neither readily available nor affordable in Africa. The Bassini technique for repair of inguinal hernias has therefore remained the standard of care in Africa [4]. According to Maggiore and colleagues in Cameroon, the Bassini procedure was recommended for hernia repair in patients under 40 years while the Lichtenstein technique was recommended for those above 40 years [13].

Once the diagnosis of an ectopic inguinal ovary is made, prompt surgical intervention and hernia repair to salvage the ovary is recommended [6] to prevent imminent strangulation, which would rather require an oophorectomy [14]. Patient follow up is of vital importance for assessment of fertility and complications such as development of recurrent ovarian cysts and chronic pain [15]. In women of reproductive age with an otherwise normally developed genitourinary system, fertility is usually conserved in the absence of complications [16]. Our patient was a sexually inactive 16-year-old girl; hence, viability of the affected ovary was confirmed mostly via ultrasonographic features of normal vascularization and cyclical changes.

## Conclusions

We have presented a rare occurrence in the African literature of an ectopic inguinal ovary in a girl, initially misdiagnosed for an acute appendicitis. Special attention should be paid when examining obese girls and women presenting with a RIF pain especially in emergency settings, as obesity in our patient contributed in a missed diagnosis. Clinicians should endeavor to consider a broader perspective of differential diagnoses when faced with girls and women of reproductive age presenting with RIF pain. Appropriate imaging may, however, be particularly helpful in uncertain circumstances. Urgent and careful surgical exploration of the hernia sac remains paramount.

## Abbreviations

BMI: Body Mass Index
CBC: Complete blood count
CRP: C-reactive protein
RIF: Right iliac fossa
